# P2X7 Receptor Signaling in Stress and Depression

**DOI:** 10.3390/ijms20112778

**Published:** 2019-06-06

**Authors:** Deidiane Elisa Ribeiro, Aline Lulho Roncalho, Talita Glaser, Henning Ulrich, Gregers Wegener, Sâmia Joca

**Affiliations:** 1 Department of Biochemistry, Chemistry Institute, University of Sao Paulo, Sao Paulo SP 05508-000, Brazil; tali.gla@gmail.com (T.G.); henning@iq.usp.br (H.U.); 2 Department of Pharmacology, School of Medicine of Ribeirao Preto, University of Sao Paulo, Ribeirao Preto 14049-900, Brazil; aline.roncalho@gmail.com; 3 Department of Physics and Chemistry, School of Pharmaceutical Sciences of Ribeirao Preto, University of Sao Paulo, Ribeirao Preto 14040-903, Brazil; 4 Translational Neuropsychiatry Unit (TNU), Department of Clinical Medicine, Aarhus University, DK-8240 Risskov, Denmark; wegener@clin.au.dk; 5 AUGUST Centre, Department of Clinical Medicine, Aarhus University, DK-8000 Aarhus C, Denmark; 6 Aarhus Institute of Advanced Studies (AIAS), Aarhus University, DK-8000 Aarhus C, Denmark

**Keywords:** P2X7 receptor, depression, stress, neuroinflammation

## Abstract

Stress exposure is considered to be the main environmental cause associated with the development of depression. Due to the limitations of currently available antidepressants, a search for new pharmacological targets for treatment of depression is required. Recent studies suggest that adenosine triphosphate (ATP)-mediated signaling through the P2X7 receptor (P2X7R) might play a prominent role in regulating depression-related pathology, such as synaptic plasticity, neuronal degeneration, as well as changes in cognitive and behavioral functions. P2X7R is an ATP-gated cation channel localized in different cell types in the central nervous system (CNS), playing a crucial role in neuron-glia signaling. P2X7R may modulate the release of several neurotransmitters, including monoamines, nitric oxide (NO) and glutamate. Moreover, P2X7R stimulation in microglia modulates the innate immune response by activating the NLR family pyrin domain containing 3 (NLRP3) inflammasome, consistent with the neuroimmune hypothesis of MDD. Importantly, blockade of P2X7R leads to antidepressant-like effects in different animal models, which corroborates the findings that the gene encoding for the P2X7R is located in a susceptibility locus of relevance to depression in humans. This review will discuss recent findings linked to the P2X7R involvement in stress and MDD neuropathophysiology, with special emphasis on neurochemical, neuroimmune, and neuroplastic mechanisms.

## 1. Introduction

Stress exposure is considered to be the main environmental cause of mental health disorders, such as major depressive disorder (MDD) [1,2,3]. MDD is a chronic condition estimated to affect more than 350 million people worldwide [4], especially individuals between 25 and 54 years old, i.e., the most productive age group [5]. Moreover, the World Health Organization (WHO) expects depression to become the leading cause of disability worldwide in 2030 [6], being associated with an increased risk of all-cause mortality and a reduced life expectancy [7,8], thus representing a major contributor to the overall global burden of disease. Importantly, a recent global return on investment analysis estimated that appropriate treatment of depressive disorders could significantly improve health quality and decrease the growing economic burden attributed with the disease [9]. Therefore, depression has a high social and economic impact, as shown by previous studies [10,11], which highlights the importance of developing appropriate diagnostic tools and new treatments.

Clinical diagnoses of MDD is based on symptom identification, with no biological marker currently available. Patients present variable symptomatology, such as weight loss or gain, inability to sleep or oversleeping, psychomotor agitation or retardation [12], making a proper diagnosis difficult. Moreover, most antidepressants induce therapeutic effects only after 3 or 4 weeks of continuous treatment [13,14], while also inducing several side effects [15]. Considering that the antidepressants clinically available so far primarily affect monoaminergic signaling, especially through inhibition of the reuptake of serotonin (5-hydroxytryptamine, 5-HT) and noradrenalin (NA), research on alternative molecular targets beyond monoamines may help to identify biological markers for the disease, as well as pointing to novel mechanisms for the development of drugs with better therapeutic profiles.

**Figure 1:**
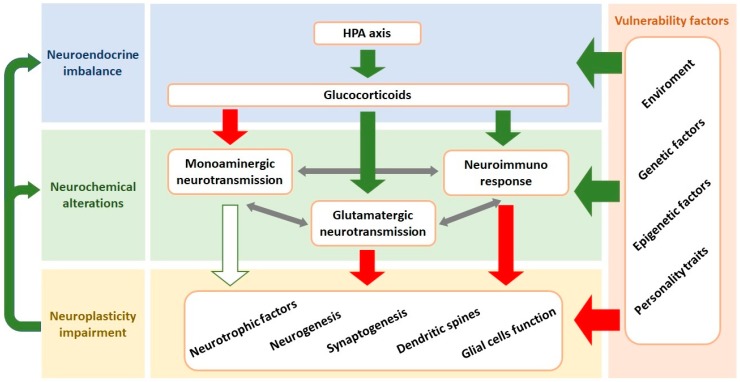
Interaction between the components involved in the neurobiology of MDD. Vulnerability factors can lead to development of MDD due to induction of changes in the central nervous system including the neuroendocrine homeostasis, neurochemical alterations, and neuroplasticity impairment. The core response to stress is the HPA axis activation resulting in high circulating levels of glucocorticoids. This endocrine response leads to neuroimmune activation, inhibition of monoaminergic and facilitation of glutamatergic neurotransmissions. These systems interact, and the overall result is impairment of the neuroplasticity in cortical and limbic structures. Neuroplasticity alterations include decreased levels of neurotrophic factors (e.g., BDNF), dendritic atrophy, diminished neurogenesis and synaptogenesis and glial cells dysfunction. Impaired neuroplasticity further contributes to neurochemical imbalances. Green arrows indicate stimulation while red arrows represent inhibition. White arrow with green border means that a stimulating action was blocked. HPA: hypothalamic-pituitary-adrenal; BDNF: brain-derived neurotrophic factor.

Evidence from recent decades has revealed that the neurobiology of depression is significantly more complex than initially proposed when the ‘catecholamine hypothesis of depression’ was proposed [16]. Current hypotheses propose that MDD results from the interplay and interaction between genetic and environmental factors, leading to neuroendocrine imbalance, neurochemical alterations (including impaired monoaminergic neurotransmission, enhanced glutamate release and neuroimmune response), as well as decreased neuroplasticity (e.g., synaptogenesis and neurogenesis) [1,17] (Figure 1).

The P2X7 receptor (P2X7R) is an ion channel activated by high concentrations of ATP, which for example can be observed following stress exposure [18]. The P2X7R has been involved in several process observed in MDD, such as impaired monoaminergic neurotransmission [19,20,21], increased glutamatergic neurotransmission [22], stimulation of a neuroinflammatory response [23] as well as reduced neuroplasticity [20,24]. In this scenario, the P2X7R becomes an interesting target for the understanding of depression neurobiology and treatment. Based on these facts, we herein present a critical review of the current evidence associating the P2X7R with stress and depression, with the overall aim to discuss an integrative view of putative underlining neurochemical and molecular mechanisms.

## 2. Overview of P2 Receptor-Mediated Signaling in the Brain

**Figure 2:**
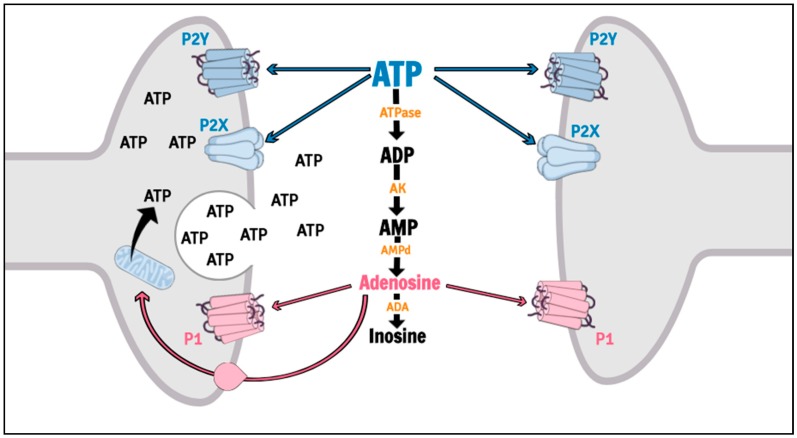
Purinergic signaling. ATP is synthesized in terminal nerves, glial cells or astrocytes by mitochondrial oxidative phosphorylation, and released to extracellular space after physiological or pathological stimulus by vesicular exocytosis, transmembrane channels (pannexin, connexin) or cellular apoptosis. Extracellular ATP may either interact with P2 receptors or be rapidly metabolized to adenosine. P2 receptors are divided in P2X (cation ionotropic) and P2Y (metabotropic) receptors. Adenosine molecules may interact with P1 receptors (metabotropic), they can be reuptaken and converted back into ATP in cell cytoplasm or they can be metabolized by ADA into inosine. ATP: adenosine triphosphate; ATPase: adenosine triphosphatase; AK: adenylate kinase; AMPd: adenosine monophosphate deaminase; ADA: adenosine deaminase.

Purinergic signaling is involved in several psychiatric disorders, including MDD [25,26,27,28], and is mediated by nucleoside and nucleotide actions on P1 (P1R) and P2 (P2R) receptors. P1R are G-protein coupled receptors activated by adenosine, while P2R can be divided in metabotropic (P2Y receptors, P2YR) and ionotropic (P2X receptors, P2XR) cation subtypes, which both is sensitive to adenosine triphosphate (ATP), adenosine diphosphate (ADP), uracil triphosphate (UTP), uracil diphosphate (UDP) and UDP-glucose [29,30]. The actions of the Purines are efficiently controlled by an extracellular enzymatic chain collectively called ectonucleotidases [31,32]. Among the purinergic receptors, the P2X7R has been suggested as a putative target for therapeutic intervention in mood disorders [33] (Figure 2).

The P2X7R, formerly known as the P2Z receptor [34], is an ATP-gated cation channel (for a detailed review, see [18]). ATP is synthesized through oxidative phosphorylation in mitochondria localized in terminal nerves, glial cells or astrocytes [35], and is found in the cell cytoplasm, stored in vesicles alone or with other neurotransmitters [36,37]. Release of ATP into the extracellular space occurs rapidly after physiological or pathological stimulus (hypoxia, hypoglycemia, ischemia, inflammation, cellular injury or stress) [38] and may be mediated by vesicular exocytosis [39,40], transmembrane channels (pannexin, connexin) [41,42], cellular apoptosis [43] or through P2X7R [44,45]. Interestingly, P2X7R have only low affinity for ATP (>100 μM), requiring exceptionally high concentrations in order to be activated, which can be observed during conditions of 2stress [46].

A recent debate has questioned the expression of P2X7R in neurons [47,48]. Despite being detected in glial and neuronal cells [49,50,51,52,53,54], the activity of the P2X7R was not always investigated and used antibodies lack specificity [49,55,56,57]. In addition, even few glial cells in the preparation studied could be responsible for the P2X7R-mediated effects firstly attributed to neurons [58,59]. On the other hand, the characteristic response following stimulation of this receptor has been demonstrated in cultured neurons or synaptosomes by electrophysiological recordings, intracellular Ca^2+^ measurements and activation of intracellular signaling cascades [50,60,61,62,63,64], which supports the hypothesis of P2X7R expression in neurons. However, further in-depth studies are required to elucidate whether these responses are reproducible in vivo.

The P2X7R is widely distributed in different brain regions, found both in neurons and in glial cells [49,55,65]. P2X7R-mediated effects, which mainly occurs through activation of the neuroinflammatory response, have been involved in MDD, neurodegenerative diseases, schizophrenia, epilepsy, neuropathic pain and brain injury [28,66,67,68]. This receptor also interferes with other mechanisms associated to stress response and the neurobiology of depression, which are supported by human and pre-clinical studies that are further described below.

## 3. Targeting P2X7 Receptor in Stress and Depression

### 3.1. Human Studies

The human P2X7R gene is located on the chromosomal region 12q24.31, a region related to bipolar disorder (BD) and depression [69,70]. Lucae et al. (2006) were the first to show the association of the non-synonymous coding single nucleotide polymorphisms (SNP) rs2230912 in the P2X7R gene with MDD [71]. The polymorphism causes an amino acid exchange from glutamine to arginine at position 460 (Gln460Arg) at long intracellular C-terminal domain of P2X7R, which have been suggested to affect both Ca^2+^ influx, P2RX7dimerization, and other protein—protein interactions having effects upon P2RX7-mediated signaling [71]. Subsequent studies, however, have shown controversial results on this polymorphism, and in a case-control study no difference in the presence of SNP rs2230912 between patients suffering from MDD and controls was shown, although it was found that the severity of symptoms of depression was directly related to the presence of the polymorphism [72]. As mentioned, the association between the severity of depressive symptoms and polymorphisms in the P2X7R gene was also demonstrated in patients diagnosed with BD [73]. Corroborating the primary study of Lucae and coworkers, it was later found that SNP rs2230912 is associated with mood disorders and longer episodes of depression [74]. Finally, two independent meta-analysis studies conducted showed controversial evidence. The first reported no association of rs2230912 polymorphism and mood disorder (MDD and BD) [75], while the latter one reported a positive association with rs2230912 polymorphism and MDD [76]. However, as discussed in the latter study [76], it is noteworthy that the included studies differed between the two metaanalyses, with the latter more recent one including more validated studies. Nevertheless, these results highlight the need for further studies regarding the presence and participation of this polymorphism in mood disorders.

### 3.2. Pre-Clinical Studies

Since the studies in humans are scarce and inconclusive, the results obtained in vitro or in animal models have provided important information regarding the role of P2X7R in stress responses and depressive disorders. Specifically, regarding the controversy on the association of Gln460Arg polymorphism in the P2X7R gene with MDD as discussed above, recent data show that mice expressing human P2X7R, with either normal (hP2X7R-wild type) or altered gene (hP2X7R-Gln460Arg), present with no behavioral changes or alterations in P2X7R activity [77]. Interestingly, heterozygous mice for both variants exhibited attenuated function of P2X7R and impaired sleep, which was also observed in healthy heterozygous human subjects [77]. Finally, hP2X7R-Gln460Arg mice show increased vulnerability to chronic social defeat stress. These results indicate that heterozygotic individuals may be more susceptible to development of depression through interaction between genetic predisposition and stress exposure [77]. In this context, it is relevant to mention that the effect of stress or antidepressant treatment on the expression and function of the P2X7R also provides supportive evidence for the involvement of the P2X7R in the neurobiology of depression and mechanisms underlying clinical antidepressant effects. Thus, the effect of antidepressants on P2X7R function has been demonstrated in whole-cell patch-clamp studies, in which paroxetine but neither fluoxetine nor desipramine administration reduced the inward currents evoked by 3’-O-(4-benzoyl)benzoyl-ATP (BzATP) on cloned rat P2X7R, which was stably expressed in human embryonic kidney 293 (HEK293) cells [78]. In another study, antidepressants demonstrated differential effects on human P2X7R-mediated responses, as paroxetine inhibited while fluoxetine and clomipramine potentiated ATP-induced dye uptake in HEK-293 cells expressing recombinant human P2X7R [79]. These results suggest that the activity of the P2X7R can be selectively modulated by different antidepressants drugs, pointing to unknown mechanisms by which these drugs may exert their therapeutic and/or side effects.

This hypothesis is further supported by evidence from in vivo animal studies. For instance, exposure of mice to chronic unpredictable mild stress (CUMS) enhanced P2X7R expression in the hippocampus and medial prefrontal cortex [80], and exposure of mice to chronic restraint stress (CRS) increased hippocampal P2X7R levels [81]. Moreover, the antidepressant-like effect induced by clemasine [80] and ketamine [81] were associated with decreased hippocampal P2X7R levels in the in stressed mice. Similarly, we recently observed that the antidepressant-like effects induced by repeated treatments with imipramine or desipramine attenuated P2X7R levels in the ventral hippocampus of rats exposed to a paradigm of inescapable foot shocks [82].

Although the aforementioned evidence indicates that antidepressants may attenuate P2X7R signaling, there are studies pointing to a more complex scenario. In studies using immunohistochemistry, no alterations in hippocampal P2X7R levels were detected in animals exposed to chronic unpredictable stress (CUS) [53]. Moreover, restraint stress was observed to reduce the expression of P2X7R in rats submitted to three different protocols: (i) acute stress (1 session of restraint stress), (ii) chronic stress (21 sessions of restraint stress, 1 per day) and (iii) chronic stress with recovery (21 sessions of restraint stress, 1 per day followed by 7 days of no stress) [83]. Interestingly, a comparative analysis of normalized data indicates that the magnitude of the P2X7R reduction was significantly greater during chronic stress compared to the acute stress group, with a gradual normalization of the expression of P2X7R in animals that were allowed to recover for 7 days following stress sessions [83]. The divergent data may be explained by the use of different techniques to identify P2X7R levels (western blotting versus immunohistochemistry). In this context, it could be relevant to characterize the regional P2X7R levels, and how the P2X7R levels might vary in the different cell types of the brain, in response to acute and prolonged stress. Association of the P2X7R levels with stress susceptibility/resilience could reveal novel important information regarding its involvement in depression neurobiology. Nevertheless, as summarized in Table 1, there seems to be consistent evidence that hippocampal P2X7R expression is modulated by stress exposure and antidepressant treatment.

The direct modulation of P2X7R expression and function in the brain by means of genetic or pharmacological manipulation, respectively, has provided additional evidence that increased P2X7R signaling might be involved in depressive-related behaviors. Basso and co-workers investigated for the first time the behavior of P2X7R knockout (KO) mice in two tests predictive for antidepressant effects, the forced swim test (FST) and the tail suspension test (TST) [84]. These mice present increased active behaviors in these tests, without changes in spontaneous locomotor activity, which can be interpreted as less depressive-like phenotype, suggesting an antidepressant-like effect of the genetic manipulation [84]. Further supporting this suggestion was found in a study with P2X7R KO mice, which showed improved response to a sub-effective dose of the antidepressant drug imipramine in the FST [84]. Additional studies further corroborated these initial findings, with observations that P2X7R KO mice presents reduced immobility time in both the FST [85] and the TST [20,85].

In another study, however, the changed behavioral phenotype of P2X7R KO mice was only observed when mice were repeatedly exposed to the FST, indicating that absence of P2X7R-mediated signaling can facilitate behavioral adaptation upon repeated stress exposure, preventing development of depressive-like behaviors, following a single exposure to the stressful condition [86]. According to the authors, repeated stress is suggested to be the key in revealing the less depression-like phenotype of P2X7R KO mice in the FST.

Altogether, data from P2X7R KO mice indicates that absence of the P2X7R results in increased resilience to stress, and a phenotype showing less depression-like characteristics. Evidence from pharmacological studies have further corroborated this hypothesis, since treatment with P2X7R antagonists induce antidepressant-like effects in various animal models. Initial evidence from Csolle et al. showed that treatment with Brilliant Blue G (BBG), a P2X7R antagonist, attenuated the anhedonia induced by lipopolysaccharide (LPS) [20] and decreased the immobility time of mice exposed to TST [85]. Similarly, our research group observed that the treatment with the non-selective P2R antagonist (pyridoxal-phosphate-6-azophenyl-2’,4’-disulfonic acid, PPADS) or with a preferential P2XR antagonist (iso-PPADS) decreased the immobility time of mice undergoing FST [87].

In line with these observations, systemic treatment with BBG (50 mg/kg, per day) or fluoxetine (15 mg/kg, per day) during 7 weeks in mice exposed to CUMS reversed the behavioral alterations induced by the chronic stress exposure, strongly suggesting an antidepressant-like effect of both interventions in the model [88]. In addition, administration of a more selective P2X7R antagonist, A-804598 (5 mg/kg, twice daily), during 28 days, also reversed anhedonia caused by CUMS [23].

Similar findings were obtained using another animal model of depression, based on selective breeding, the flinders sensitive line (FSL) rats [89,90], where seven days treatment with A-804598 (30 mg/kg, i.p., per day) induced antidepressant-like effects in the FST [82]. However, in another study, administration of A-804598 (25 mg/kg) to rats through intubation failed to reverse behavioral changes caused by foot shocks [91]. The divergent results may be explained by the different doses and administration routes of A-804598 in each study.

As systemic injections have limited capacity to reveal the role of distinct brain regions, studies with intracerebral administrations have been carried out. It was found that microinjection with two P2X7R antagonists, BBG (1 pM) and A-438079 (1.75 nM), into the rat hippocampus, during 3 weeks, prevented the development of depressive-related behaviors induced by daily exposure to CUMS, pointing to hippocampus as an important site where the P2X7R might regulate behavioral/emotional responses to stress [53]. In line with these observations, hippocampal administration of P2R agonists (ATP 100 nmol/rat or BzATP 10 nmol/rat) for 3 weeks resulted in depressive-like behaviors similar to those caused by stress exposure [53]. It can therefore be hypothesized that stress may trigger excessive ATP release, followed by activation of P2X7R signaling in the hippocampus. This may subsequently impair mechanisms involved in behavioral adaptation to chronic aversive situations, and facilitate the development of depressive-like behaviors. While this points to the importance of the hippocampus, the involvement of other brain regions remains to be explored.

Altogether, both pharmacological and genetic evidence strongly suggest that P2X7R stimulation during stress increase the vulnerability and subsequent development of behavioral adversities associated with the neurobiology of depression (Table 2).

## 4. Mechanisms Regulated by P2X7 Receptor Signaling with Relevance to Stress and Depression

### 4.1. Neurochemical Mechanisms

P2X7R is thought to play an important role in modulating the release and uptake of different neurotransmitters dysregulated in stress and depression, such as 5-HT, NA and glutamate. Therefore, the P2X7R might have an important role in controlling brain neurochemistry under stressful circumstances.

Although there is scarce evidence regarding the localization of P2X7R in monoaminergic terminals, studies conducted with radiolabeled ligands indicate that activation of presynaptic P2R in rat cortical slices inhibits electrically-stimulated 5-HT release [94]. However, the specific purinergic receptor subtype involved in this effect was not characterized, due to the lack of selective drugs. However, a role for P2X7R in regulating 5-HT release has been confirmed in more recent studies with genetic and pharmacological approaches, where it was reported that median raphe nuclei stimulation decreased the 5-HT release in the hippocampus of genetically deficient in P2X7R mice, and after perfusion with selective antagonists (JNJ-47965567 or AZ-10606120) [19]. In another study, however, P2X7R KO mice presents with increased hippocampal 5-HT levels, increased 5-HT transporter binding sites and 5-HT uptake, along with behavioral adaptation to stress [20]. As it is well documented that the median raphe nuclei are activated in response to stress, increased 5-HT release in the hippocampus to promote stress adaptation is a notable consequence [92,95]. Based on these findings, P2X7R-mediated increase in 5-HT levels might be an important mechanism involved in behavioral adaptation to stress.

P2XR stimulation also elicits transporter-mediated NA release from rat hippocampal slices [21], although the physiological relevance of this mechanism is not yet fully understood. In a recent study, it was shown that the antidepressant-like effect in mice induced by systemic treatment with PPADS (P2R inhibitor) was blocked by 5-HT or NA depletion, implicating these monoamines in the drug effect [96]. Indeed, co-treatment with sub-effective doses of PPADS and serotonergic (fluoxetine) or noradrenergic (desipramine) antidepressants had synergistic effects [96]. Based on that, it is plausible to suggest that P2X7R stimulation could inhibit 5-HT and NA release upon stress exposure and impair behavioral adaptation. Similarly, blockade of P2X7R-mediated signaling could disinhibit 5-HT and NA release in limbic brain regions and promote stress coping behaviors. This hypothesis, however, needs further investigation, and additional studies are required to better elucidate the neural pathways involved in this response under conditions of acute and repeated stress.

Interestingly, NA may trigger ATP release from glial cells activating P2X7R, subsequently enhancing the efficacy of glutamatergic neurotransmission, by postsynaptically facilitating α-amino-3-hydroxy-5-methyl-4-isoxazolepropionic acid (AMPA) receptor insertion [97]. This is in line with studies showing that genetic deletion of P2X7R in mice inhibits glutamate release and downregulates AMPA and metabotropic glutamate receptor subunits and upregulates the NR2B subunit of N-methyl-D-aspartate (NMDA) receptors in the amygdala of P2X7R deficient mice [20]. Gamma-amino butyric acid (GABA) release modulation by these receptors has also been documented. Dual labeling immunoreactivity studies in rats indicate that P2X7R may be co-localized in glutamatergic and GABAergic terminals [98]. Moreover, P2X7R stimulation by ATP analogues elicited a concentration-dependent GABA efflux from rat hippocampal slices, which was reduced by treatment with the voltage-dependent sodium (Na^+^) channel blocker (tetrodotoxin, TTX) or kainate receptor antagonists (gadolinium or 6-cyano-7-nitroquinoxaline-2,3-dione, CNQX), but not with the NMDA antagonist (AP-5) or AMPA receptor blocker (GYKI 53655) [51]. P2X7R antagonists (BBG or PPADS) reduced GABA release elicited by both ATP and electric stimulation [51]. Altogether, these results suggest that stimulation of the P2X7R in hippocampal nerve terminals induce glutamate release, which may leads to kainate receptor-mediated GABA efflux from interneurons [51]. In accordance with these results, glutamate and GABA release induced by both electrical stimulation or P2X7R activation were absent in hippocampal slices of the P2X7R KO mice [99]. In addition, the amplitude of glutamate-evoked currents recorded from neurons of cortical slices, were slightly decreased by both genetic (KO mice) or pharmacological (JNJ-47965567) inhibition of the P2X7R [100].

Most of the aforementioned effects were attributed to the stimulation of P2X7R expressed in nerve terminals. However, as these analyses were performed in brain slices, in which glial cells were also present in the preparation, it cannot be excluded that P2X7R activation in glial cells also could have elicited the observed responses [101]. Another issue is that exogenously applied P2R agonists may be hydrolyzed to adenosine [102], which is known to induce glutamate release [103,104,105]. The effects mediated by P2X7R in glial cells and adenosine can be avoided in studies in pure neuronal cell cultures [106] and synaptosomes [107,108,109], which confirms that stimulation of P2X7R expressed in nerve terminals favors glutamate and GABA release. However, participation of P2X7R localized in glial-cells on the net effect on glutamate release has also been indicated by radiolabeled studies in murine cortical astrocyte cultures [110] and whole-cell patch-clamp analysis [111]. P2X7R stimulation decreased GABA and glutamate uptake by nerve terminals from rat and human cerebral cortex [112,113] as well as glutamate uptake and glutamine synthetase activity in astrocyte-cultures [114]. Finally, P2X7R activation reduced expression of glutamate/aspartate sodium-dependent transporter (GLAST), a mechanism directly involved in the extracellular clearance of glutamate by astrocytes [115].

Further studies are required to clarify the role of P2X7R in the tripartite synapse and detailed distribution in the central nervous system (CNS). However, regardless of their specific location, P2X7R stimulation increase the release of glutamate and GABA, as well as decrease the uptake/clearing of these transmitters. Importantly, increased glutamate levels has been associated with mechanisms involved in stress and depression, such as neuro-inflammatory process, decreased levels of neurotrophic factors, diminished neurogenesis, and neuroplasticity impairment [116,117]. These processes are also regulated by P2X7R.

As increased glutamate levels are observed in the brains of animals exposed to stress [118,119] and in depressed individuals [120,121], understanding the importance of the regulation of glutamatergic neurotransmission in stress and depression are crucial. Interestingly, antidepressants are believed to exert one of the therapeutic effects by modulating the extracellular availability of this neurotransmitter [122,123,124,125]. For example, in humans, a single intravenous injection of the NMDA receptor antagonist, ketamine, induce an antidepressant effect within 4 h lasting up to 7 days [126]. Recently, the intra-nasal administration of ketamine was approved for treatment resistant-depression [127]. The neurobiological effects underlying this remarkable effect have not been clarified in detail, but are likely to involve multiple biological substrates, pathways and circuits.

Despite the evidence discussed above, it is not yet known to what extent glutamate or GABA release modulated by P2X7R stimulation contributes to the behavioral responses elicited by stress exposure. Quantification of glutamate and GABA levels in different brain regions of animals exposed to stress and treated with P2X7R antagonists is needed in order to clarify this issue. Moreover, the effects of direct pharmacological modulation of glutamatergic and GABAergic receptors in P2X7R KO animals, or in animals treated with P2X7R antagonists could also reveal the importance of such mechanisms in P2X7R-mediated signaling during stress.

Beyond 5-HT, NA, glutamate and GABA, P2X7R stimulation may also modulate nitric oxide (NO) formation. Our research group observed that systemic administration of PPADS or iso-PPADS induced antidepressant-like effect in mice associated with decreased NO levels in prefrontal cortex [87]. Besides, a molecular analysis suggest that P2X7R may be coupled to neuronal nitric oxide synthase (nNOS) in prefrontal cortex [87]. Indeed, a double immunofluorescence study have shown that P2R are co-localized with nNOS in some brain regions, including the hippocampus [128]. These results indicate that P2X7R may also modulate stress-induced consequences by facilitating NO release in limbic brain regions. This is coherent with evidence that inhibition of NO synthesis induces antidepressant-like effects (for a review, see reference [129]).

Altogether, there are substantial evidence that P2X7R modulates 5-HT, NA, glutamate, GABA and NO release, which are mechanisms consistently involved in stress and depression [123,129,130]. However, the precise mechanisms by which P2X7R regulates this neurochemical balance during stress is not yet known and deserves further investigation.

### 4.2. Neuroinflammatory Response and Inflammasome Activation

**Figure 3:**
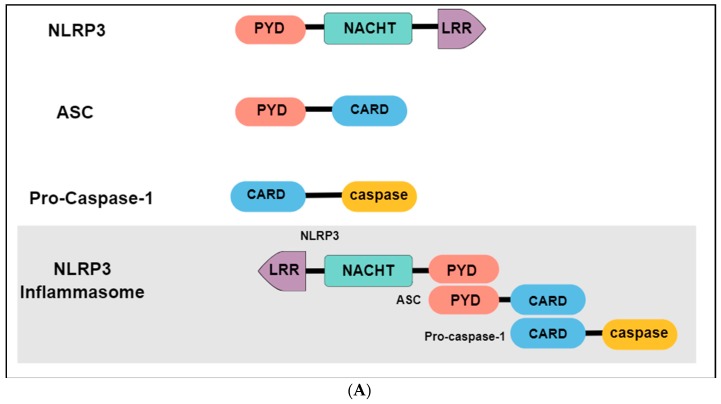

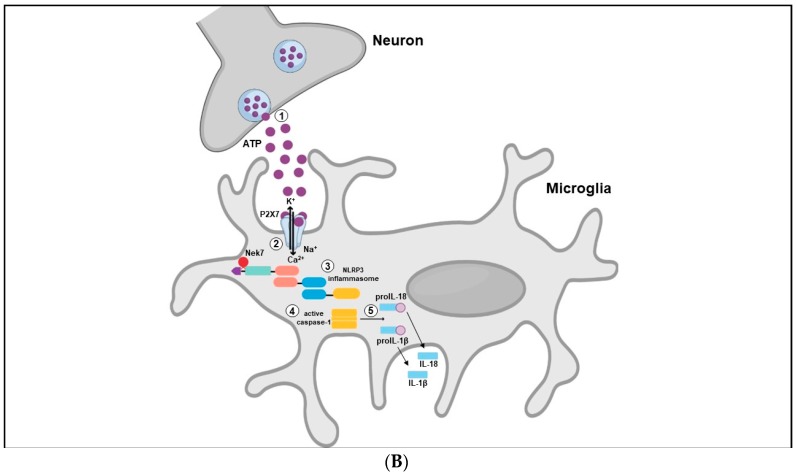
P2X7R-mediated NLPR3 inflammasome activation. (**A**) NLRP3 is composed by an N-terminal pyrin domain (PYD), a central nucleotide-binding-and-oligomerization (NATCH) domain, and a C-terminal leucine-rich repeats (LRR) domain. PYD domain recruits the apoptosis-associated speck-like protein (ASC) that contains a caspase recruitment domain (CARD). This complex recruits the procaspase-1. Together, these components constitute the NLRP3 inflammasome. (**B**) 1. High ATP levels released from neurons or astrocytes reach P2X7R located in microglia; 2. P2X7R stimulation elicits K^+^ efflux, which may trigger NLRP3 inflammasome assembly and activation through NIMA-related serine/threonine kinase 7 (Nek7) binding; 3. NLRP3 inflammasome mediates the activation of caspase-1; 4. Caspase-1 induces the maturation of interleukins (IL) by cleaving pro-IL-1β and pro-IL-18 in IL-1β and IL-18, respectively; 5. Finally, the mature form of cytokines are secreted.

The immune system may present two different responses when challenged by a threat stimulus: activation of innate or adaptative immunity. The innate immune response consists of the recognition of pathogen-associated molecular patterns (PAMPs) or damage-associated molecular patterns (DAMPs) by pattern-recognition receptors (PRRs), expressed in innate immune and inflammatory cells such as macrophages, microglia, monocytes, neutrophils and dendritic cells [131]. Among the different types of PRRs, the newest described is the inflammasome, a high-molecular-weight complex, present in the cytosol of immune cells [132]. The most studied and best-characterized inflammasome is the NLR family pyrin domain containing 3 (NLRP3), which belongs to the nucleotide-binding oligomerization domain (NOD) - like receptors. Structurally, the NLRP3 inflammasome presents a tripartite domain organization: a C-terminal domain with regulatory functions composed by leucine-rich repeats (LRR), a nucleotide binding-and-oligomerization domain (NACTH) centrally located that regulate the self-oligomerization, and an N-terminal pyrin domain (PYD) that recruits an adaptor molecule called apoptosis-associated speck-like protein containing a caspase recruitment domain (ASC or PYCARD) (Figure 3A) [133]. The activation and assembly of the inflammasome begin with the release of PAMPs and DAMPs, causing an opening in NLRP3, which allows the interaction between the PYD domain in NLRP3 and ASC. Subsequently, the caspase recruitment domain (CARD) of ASC binds to the CARD of procaspase-1, originating the NLRP3 inflammasome. Formation of this complex elicit the self-cleavage of procaspase-1, generating the active caspase 1 and inducing the conversion of proinflammatory cytokines such as interleukin 1 beta (IL-1β) and interleukin 18 (IL-18) in their active forms, that will be secreted from activated immune cells (Figure 3B) [134,135].

Since the observation that depressed patients have changes in the immune system [136,137], neuroimmune mechanisms have been seen as a key mechanism to the development of depression, and the molecular components of neural circuits involved in these processes have been increasingly investigated. Increased levels of pro-inflammatory cytokines such as interleukin (IL)-1β, IL-6, IL-18, tumor necrosis factor-α (TNF-α) and interferon-γ (IFNγ) have been consistently reported in the blood of depressed patients [138,139,140] and in brain regions of stressed animals [141,142,143]. Moreover, antidepressant treatment seem to attenuate this immunological response [140,143].

In a stressful situation (physical or psychological), the increase in ATP release may be interpreted as a DAMP signal by the immune system in the CNS, especially by microglial cells [144]. A clinical report published in 2014 showed that the levels of NLRP3 and caspase-1 were increased in the blood of untreated depressive patients along with increased serum levels of IL-1β and IL-18 [145]. The treatment with the tricyclic antidepressant (TCA) amitriptyline was able to reduce the levels of NLRP3, caspase-1, IL-1β and IL-18, suggesting that, in some way, antidepressants modulate the activation and/or release of inflammatory components [145]. In agreement with these findings, earlier studies have demonstrated that both TCA and selective serotonin reuptake inhibitors (SSRIs) normalize the serum levels of pro-inflammatory cytokines in patients with MDD [146,147,148] suggesting that the relationship between stress, depression and inflammation does not occur in a single direction, but that the three elements of this equation act on each other.

Studies with animal models also provide evidence to the involvement of NLRP3 in the neurobiology of depression. Mice submitted to 4 weeks of CUMS presents with higher serum levels of IL-1β, along with enhanced hippocampal NLRP3, caspase-1 and active IL-1β protein levels [149]. As expected, inhibition of the NLRP3 was able to reduce the serum and hippocampal levels of IL-1β, while attenuating the depressive-like behaviors induced by stress [149]. A functional inflammasome NLRP3 appears to be necessary for the development of depressive behaviors induced by stress, since genetic depletion of NLRP3 in mice prevented the stress-induced alterations associated with depression, like decreased sucrose preference, reduced social interaction ratio and high immobility time in the FST [150].

Interestingly, P2X7R is activated only by high concentrations of ATP (EC_50_ ≥ 100 µmol·L^−1^), which occurs after stress exposure [34]. The activated P2X7R allows Ca^2+^ and Na^+^ influx as well as potassium (K^+^) efflux from cells. Decreased K^+^ levels in the cytosolic microenvironment may lead to NLRP3 inflammasome assembly and activation through NIMA-related serine/threonine kinase 7 (Nek7) binding, which culminates in the secretion of inflammatory cytokines, such as IL-1β and IL-18 [151,152] (Figure 3B).

Another convergent point between purinergic signaling and the NLRP3 inflammasome is that P2X7R as well as NLRP3 are heavily expressed in microglia [47]. In fact, stimulation of microglia cell culture with ATP enhance the expression of NLRP3, ASC protein and caspase-1 along with enhanced secretion of IL-1β and IL-18 in a NLRP3 inflammasome-dependent manner. The same NLRP3 inflammasome components (ASC, caspase-1 and cytokines) were not observed in astrocytes culture cell, indicating a cell-type specific NLRP3 expression [153]. Additionally, the effects of ATP in microglia NRLP3 activation were blocked by pre-treatment with potassium chloride (KCl), preventing the efflux of K^+^, suggesting requirement of low concentrations of this ion in the intracellular environment for the activation of NLRP3 inflammasome [153].

Altogether, these data support the notion that increased ATP levels induced by stress exposure may stimulate P2X7R leading to diminished intracellular K^+^ concentration, with subsequent NLRP3 inflammasome activation and secretion of pro-inflammatory cytokines [93,154].

This proposal is further substantiated in a study where CUS increased ATP levels and promoted activation of the hippocampal NLRP3 inflammasomal pathway [53], while P2X7R KO mice showed a less depression-like phenotype after a challenge with LPS. These findings were associated with inability to release IL-1β [20,84]. In another study, BBG treatment blocked increase in TNF-α levels in serum and induced antidepressant-like effect in LPS-treated mice [155]. Furthermore, as already mentioned, systemic treatment during 7 weeks with BBG (50 mg/kg, per day) or fluoxetine (15 mg/kg, per day) reversed the behavioral alterations and microglial activation in cortex, hippocampus and basal nuclei of mice exposed to CUMS [88]. Similarly, acute treatment with A-804598 (10 mg/kg, i.p.) completely blocked activation of the NLRP3 inflammasome, release of IL-1β and TNF-α in hippocampus following restraint stress [23]. However, administration of A-804598 (25 mg/kg) in rats through intubation only partially attenuated the increase in IL-1β and CD14 mRNA in the paraventricular nucleus, and failed to reverse the behavioral changes induced by stress [91]. However, the dose or administration route used in this study may have not been sufficient for A-804598 reach the CNS and induce antidepressant/anti-inflammatory effects.

Interestingly, a recent study reported that the antidepressant-like effect induced by ketamine, attenuated the LPS-induced depressive-like behavior together with hippocampal over-expression of IL-1β and NLRP3 [156]. Moreover, selective inhibition of the NLRP3 inflammasome with systemic treatment using Ac-YVAD-CMK, exhibited anti-inflammatory and antidepressant effects [157,158] This study raises the possibility that antidepressant effects may be associated with the ability of drugs to down-regulate activation of the NLRP3 following exposure to chronic stressful situations. This highlights the P2X7R as an important target in promoting stress adaptation and providing potentially antidepressant effects through its ability to regulate inflammasome activation.

### 4.3. Neurogenesis and Neuroplasticity Process

Neural progenitor cells (NPCs) may generate cells of the neural lineage, neurons and glial cells by differentiation, as well as self-renewal in order to maintain the original pool of parent cells [159,160]. Neurogenesis in the adult brain may therefore takes place at the sites in which the pool of NPCs is active, called neurogenic niches. In mammals there are two canonical regions, the subventricular zone (SVZ) and subgranular zone (SGZ) at the dentate gyrus of the hippocampus [159,161]. However, other regions like neocortex, striatum, amygdala, hypothalamus, substantia nigra, cerebellum and brain stem may also have sporadic neurogenesis [162]. These regions are important for cognitive functions like learning and memory, emotion control, anxiety and stress response [163,164,165].

The differentiation process is triggered in order to replace dead cells, which for example are controlling the retrieval of memories. Therefore, quiescent NPCs exit the dormant state, and re-enter in the cell proliferation cycle. The new-born cells are then determined to neuronal vs glial fate, migrate, maturate and integrate to the neuronal circuitry [166]. Meanwhile, the new cells gain the archetypical morphology by extending projections - neurites (neuritogenesis), which further matures to dendrites and axons [167]. These new extensions will connect to existing neurons, creating new synapses (synaptogenesis) [168].

Together, neuritogenesis and synaptogenesis are main neuroplasticity processes in the CNS, which are adaptations to the surrounding environment of the organism. New neurons that are still maturing possess an intriguing capability to sense information and fine-tune synapses, promoting synaptic plasticity, leading to increased sensitivity and potentiation upon cognitive demands, environmental stimuli, and behavioral or sensory experiences [166,169]. In addition, throughout life, the brain acquires and registers information in order to better adapt to environment, inducing the establishment of new cognitive blocks, like emotional control [170].

According to the neurogenic hypothesis of depression, impaired adult hippocampal neurogenesis would be inhibited by stress, and be a causal factor for triggering depressive episodes; while antidepressant treatments would induce therapeutic effect by restoring normal hippocampal neurogenesis [171]. There are studies suggesting that decreased neurogenesis is not a major contributor to the MDD development, but it may be associated to cognitive impairments induced by stress and observed in patients with depression [172,173]. In line with this prospect, mice lacking adult hippocampal neurogenesis are not more susceptible to stress [174] or do not present more depressive-related behavior [175,176]. However, neurogenesis have been reported to be required for antidepressant response [174,175,177,178], although many of these studies are controversial and lacks replication. Moreover, the relevance of neurogenesis in adult humans is heavily debated [179,180]

Research on stress-induced consequences and antidepressant effect on neuroplasticity processes have given rise to the neuroplasticity hypothesis of depression [181,182,183]. According to that, mood alterations, cognition, learning and memory impairments induced by stress or depressive conditions are a result of dysfunctional neuroplasticity, which can be observed as affected neurogenesis and/or synaptogenesis [184]. In line with this, stress—which also is a crucial environmental factor in the pathology of depression—diminish the number of dendritic spines and synapses [185], cause dendritic atrophy [186,187] and leads to reduced glial cells (both in number and size) [188,189] in structures related to development of depression, such as hippocampus and prefrontal cortex. Furthermore, studies suggest that restoration of neuroplasticity is fundamental for antidepressant effects [190,191].

Since increased neurogenesis and synaptogenesis could reverse depressive-related behaviors induced by chronic stress, understanding these processes are crucial in the search and development for new antidepressant drug targets. As recently reviewed by our group [192], extracellular ATP plays important roles in neurogenesis through stimulation of purinergic receptors, especially P2X7R. Importantly, during embryo development, it has been reported that P2X7R expression could be detected in immature neurons of the SGZ [193] and in brain neural stem cells (NSCs) of SVZ and SGZ [194,195]. Moreover, stimulation of P2X7R induced cell death of NPCs [196], which subsequently during the development may adjust the number of neurons in the organism [197].

Interestingly, studies indicate that P2X7R expressed in cultured NPCs prepared from the adult mouse SVZ, can trigger apoptosis through activation of caspase 3, which can be of particular relevance during pathological conditions, such as stress exposure which is known to increase ATP release [198]. Therefore, P2X7R stimulation would shift the homeostasis of the NPC pool, leading to increased cell death or inhibition of neurogenesis, which could contribute to the establishment of the depressive behavior.

These suggestions are supported by results showing that P2X7R KO mice presents enhanced neurogenesis [20]. Furthermore, P2X7R KO also shows reduction in the spine synapse number in DG of hippocampus, associated with improved performance in the learned helplessness model of depression [24]. Although the mechanisms by which P2X7R controls neuroplasticity are not yet entirely understood, there is support for a claim that it might involve regulation of brain-derived neurotrophic factor (BDNF), a neurotrophin essential for neurogenesis and synaptogenesis in the developing and adult brain. BDNF has been consistently associated to the therapeutic effect of antidepressant drugs [199,200,201]. Intriguingly, administration of BzATP caused a decrease in BDNF levels, which could be reversed by BBG treatment, suggesting that this response was mediated by P2X7R [20]. In addition, it was also observed by our group that P2X7R blockade with A-804598, reverted the depressive-like behavior exhibited by FSL rats, an effect which also was associated with activation of the BDNF signaling pathway in ventral hippocampus [82].

Although emerging evidence indicates that P2X7R signaling impairs neurogenesis and synaptogenesis, both processes that are important for antidepressant effects, there is still little evidence relating the antidepressant effect induced by P2X7R antagonists with altered neuroplasticity events in the brain, especially for the prefrontal cortex and hippocampus. Specifically, it is not known how P2X7R stimulation affects BDNF levels and neuroplasticity.

## 5. Final Remarks

**Figure 4:**
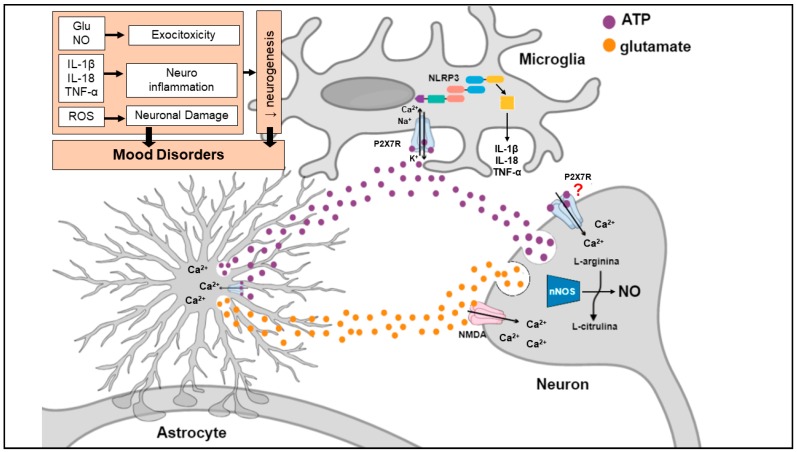
P2X7 receptor involvement in mood disorders. Stress exposure elicits a massive ATP and glutamate release with consequent activation of P2X7R and NMDA receptors, respectively. Stimulation of these receptors leads to: 1. enhanced K^+^ efflux resulting in NLRP3 inflammasome activation and secretion of inflammatory cytokines (e.g., IL-1β, IL-18, TNF-α) from astrocytes and microglia; 2. increased Ca^2+^ influx leading to ATP and glutamate release from nerve terminals and astrocytes, which is responsible to excitotoxicity; 3. NMDA-mediated nNOS activation and consequent NO formation in nerve terminals, also contributing to excitotoxicity process; 4. ROS production causing neuronal damage. Under conditions of stress, high levels of ATP, glutamate and pro-inflammatory cytokines are maintained by a regenerative circuit, even after stress stimulus termination, which leads to diminished BDNF levels, decreased synapto-/neuro-genesis and damage of brain circuits important for emotional/mood regulation. Further investigation is required to better elucidate whether P2X7R induce glutamate and ATP release by its direct action in neurons or due the indirect activation of these receptors in glial cells. ATP: adenosine triphosphate; Ca^2+^: calcium; IL-18: interleukin-18; IL-1β: interleukin-1β; K^+^: potassium; Na^+^: sodium; NMDA: N-methyl-D-aspartate receptor; nNOS: neuronal nitric oxide synthase; NO: nitric oxide, ROS: reactive oxygen species; TNF-α: tumor necrosis factor alpha.

Stress exposure, the main environmental cause of MDD, elicits a massive ATP and glutamate release from neurons and glial cells with subsequent stimulation of the P2X7R and NMDA receptor (Figure 4). Stimulation of these receptors leads to: 1. enhanced K^+^ efflux resulting in NLRP3 inflammasome activation and secretion of inflammatory cytokines (e.g., IL-1β, IL-18, TNF-α) from astrocytes and microglia; 2. increased Ca^2+^ influx leading to ATP and glutamate release from nerve terminals and astrocytes, which is responsible to excitotoxicity; 3. NMDA-mediated nNOS activation and subsequent NO formation in nerve terminals, also contributing to excitotoxicity process; 4. ROS production causing neuronal damage. Under condition of stress, high levels of ATP, glutamate and pro-inflammatory cytokines are maintained by a regenerative circuit, even after stress stimulus termination, leading to diminished BDNF levels, decreased synapto-/neuro-genesis and damage of brain circuits important for emotional/mood regulation.

Further investigation is required to better elucidate whether P2X7R modulates the function of the tri-synaptic networks through its direct action in neurons, or via the indirect stimulation of these receptors in glial cells. Additionally, characterization of the molecular and neurochemical mechanisms involved in P2X7R signaling during stress could provide important information regarding the neurobiology of depression and antidepressant effects.

P2X7R has been proposed to be a potential target for therapeutic intervention in mental disorders, due to its activity in neuroinflammatory processes, which are significantly involved in MDD, schizophrenia, epilepsy and neurodegenerative diseases (e.g., multiple sclerosis, Huntington, Parkinson’s and Alzheimer’s disease) [66,67,68]. Hence, brain penetrant P2X7R antagonists have been developed, and testing the clinical effects of these drugs in neuropsychiatric disorders is the next step [67,68]. The use of P2X7R antagonists as monotherapy or adjunctive with other drugs for treating mental disorders would benefit patients with high inflammatory disease burden, which may not respond to current available treatments.

Finally, given the high incidence of patients resistant to conventional monoaminergic antidepressants, targeting P2X7R might represent a promising approach in the search for more effective antidepressants in the clinical practice, since it re-establishes both neurochemical and neuroimmunological mechanisms that are compromised in depression.

## Figures and Tables

**Table 1:** P2X7R modulation by stress and antidepressant treatment.

Stress/Treatment	P2X7R	Cell Type/Brain Structure	Analysis Technique	References
paroxetine	↓ BzATP-evoked inward currents	cloned rat P2X7R expressed in HEK 293 cells	Whole-cell patch-clamp	[78]
fluoxetine or desipramine	no change			
paroxetine	↓ ATP-induced dye uptake	recombinant human P2X7R expressed in HEK-293 cells	dye uptake assay	[79]
fluoxetine or clomipramine	↑ ATP-induced dye uptake			
inescapable foot shocks	no change	ventral hippocampus of stressed rats	WB	[82]
imipramine	↓ expression			
CUMS	↑ expression	hippocampus of stressed mice	WB	[80]
clemastine	↓ expression			
chronic restraint stress	↑ expression	hippocampus of stressed mice	WB	[81]
ketamine	↓ expression			
CUS	no change	hippocampus of stressed rats	IHC	[53]
Acute or chronic restraint stress	↓ expression	hippocampus of stressed rats	IHC	[83]

ATP: adenosine triphosphate; BzATP: 3’-O-(4-benzoyl)benzoyl-ATP; HEK: human embryonic kidney; WB: western blotting; CUMS: chronic unpredictable mild stress; IHC: immunohistochemistry.

**Table 2:** Effect of P2X7R inhibition in animal models of depression.

P2X7R Modulation	Specie	Model	Effect	Mechanism Involved	References
Genetic deletion	mice	FST, TST	Antidepressant-like	IL-1β release absence.	[82]
Genetic deletion	mice	FST	Antidepressant-like effect after 3 sessions of FST, but not 1	Decreased activation of hippocampus dentate gyrus and basolateral amygdala.	[84]
Genetic deletion/pharmacological blockade (BBG)	mice	FST, TST	Antidepressant-like	Increased levels of NA in amygdala; and attenuated stress-induced ACTH and corticosterone responses.	[83]
Genetic deletion/pharmacological blockade (BBG)	mice	TST and SPT after LPS challenge	Antidepressant-like	Absence of P2RX7-mediated glutamate release, elevated basal BDNF production, enhanced neurogenesis and increased 5-HT bioavailability in the hippocampus.	[19]
Pharmacological blockade (iso-PPADS)	mice	FST	Antidepressant-like	Decreased NOS1 activation and NO synthesis in prefrontal cortex.	[85]
Pharmacological blockade (PPADS)	mice	FST	Antidepressant-like	5-HT and NA availability.	[92]
Pharmacological blockade (A-804598)	FRL/FSL rats	FST	Antidepressant-like	Activation of BDNF signaling pathway in ventral hippocampus.	[80]
Pharmacological blockade (A-804598)	rats	CUS/restraint stress	Antidepressant-like	Inhibition of inflammasome activation in the hippocampus.	[22]
Pharmacological blockade (A-804598)	rats	Foot shocks	No effect	Partially attenuated the increase in IL-1β and CD14 mRNA in the paraventricular nucleus induced by stress.	[89]
Pharmacological blockade (BBG)	mice	TST and SPT after LPS challenge	Antidepressant-like	Decreased serum levels of TNF-α in serum after LPS treatment.	[93]
Pharmacological blockade (combined hippocampal microinjection of BBG and A-438079	rats	CUS	Antidepressant-like	CUS increased ATP and NLRP3 inflammasomal activation in the hippocampus but the effect of P2RX7 blockade was not investigated.	[52]
Pharmacological blockade (BBG)	mice	CUMS	Antidepressant-like	Regulation of HPA axis and decrease microglial activation in cortex, hippocampus and basal nuclei.	[86]

5-HT: 5-hydroxytryptamine or serotonin; ACTH: adrenocorticotropic hormone; ATP: adenosine tri-phosphate; BBG: brilliant blue G; BDNF: brain-derived neurotrophic factor; CD14: cluster of differentiation 14; CUMS: chronic unpredictable mild stress; CUS: chronic unpredictable stress; FRL: flinders resistant line; FSL: flinders sensitive line; FST: forced swim test; HPA: hypothalamic-pituitary-adrenal; IL-1β: interleukin 1β; LPS: lipopolysaccharide; mRNA: messenger ribonucleic acid; NA: noradrenaline; NLRP3: NLR family pyrin domain containing 3; NO: nitric oxide; nNOS: neuronal nitric oxide synthase; P2X7R: P2X7 receptors; PPADS: pyridoxalphosphate-6-azophenyl-2’,4’-disulfonic acid; SPT: sucrose preference test; TNF-α: tumor necrosis factor-α; TST: tail suspension test.
